# Supplementary material for: Care professionals’ accounts of providing support and treatment for people with co-occurring alcohol use disorder and depression in the North East of England, UK: A qualitative study informed by complexity theory
Source: PLoS One. 2025 Oct 15;20(10):e0334524. doi: 10.1371/journal.pone.0334524 (PMC12527159; doi:10.1371/journal.pone.0334524)
Supplement: S2 File — (DOCX) [file pone.0334524.s002.docx]

**Supplementary Materials. Interview Topic Guide.**

**RESEARCH STUDY: NIHR ADEPT - ALCOHOL USE DISORDER AND DEPRESSION PREVENTION AND TREATMENT**

**PROFESSIONALS INTERVIEW TOPIC GUIDE**

# Introduction

- Thank you for taking the time to meet and share your experiences and views about how care is delivered for patients/service users with co-occurring depression and heavy drinking in the North East & North Cumbria Integrated Care System.
- Anything you share here today will be treated with confidence. The names of any places or people you mention will be changed to ensure you can’t be identified.
- Can I check that you have read the study participant information sheet, do you have any questions for me?
- Work through written consent.
- There are no right or wrong answers the interview is about your views and experiences.
- Are you ok for me to turn the recorder on and start the interview?

# Background

*First, we would like to find out about the work that your organisation does and your personal role and responsibilities.*

1. Could you tell me about your background and experience, and the role you hold at this organisation?
2. Could you tell me briefly about the services you / your organisation provide for people who are experiencing co-occurring depression and heavy drinking? *(Probe: focus of services; whether mostly depression or alcohol etc; how the service defines depression and heavy drinking, geographical/administrative remit)*

# Caring for people with co-occurring needs

*Next, we want to hear about your views on and experiences of providing services or support for people who are experiencing co-occurring depression and heavy drinking*.

1. Please could you give me an example (either anonymised or hypothetical) of how you would work providing services /support for a patient or service user with co-occurring depression and heavy drinking? *(Probe: pathway of care, perception of scale of co-occurring depression and heavy drinking in work they do)*
2. Could you tell me what you think helps or facilitates your work with people who are experiencing co-occurring depression and heavy drinking? (Probe: interventions, training, relevant guidelines, referral process,
3. On the other hand, what are the main barriers you face working with people who are experiencing co-occurring depression and heavy drinking? (Probe: interventions, training, relevant guidelines, referral process)

# Collaboration and joined-up service provision

*Now we would like to understand how you and your organisation work with other service providers in the region, and any challenges you face in doing so*

1. Can you tell me about how and when you work with other services in the NENC ICS to support people who experience co-occurring depression and heavy drinking? *(Probe: which point in the care pathway; what type of relationship/support function)*
2. Can you tell me about any health or social care services you feel you work particularly well with to support this client group? Please can you give an example of how this works? What do you think make this work well?
3. Overall, could you tell me about what you think supports effective joint working when working with this client group?
4. Are there any services where you experience barriers to joint-working to support this client group? Please can you give an example? What do you think the barriers are to working together?
5. Overall, could you tell me about what you think are the main barriers to effective joined-up working and collaboration in the region when working with this client group?
6. What do you think could be done to support joined-up working and collaboration in the region when working with this client group?

# Digital technology

*Finally, another focus of this study is to explore the potential of digital technology to improve system access for these patients or service users.*

1. What digital technologies do you currently use in your work with people who experience co-occurring depression and heavy drinking. For example, this could be directing patients/service users to online therapies or apps; sharing patient information electronically. *(Probe: digital interventions for AUD/depression, relevant electronic administrative datasets)*
2. In your experience of using digital technologies, what opportunities do you think they offer for supporting this client group? Can you give a specific example of where you feel they have worked well? *(Probe: improved or more timely access; enhance self-management; raise awareness/education etc)*
3. What, if any, concerns do you have about using digital technologies with this client group? Can you give a specific example of where you feel using digital technology has caused problems? *(Probe: impersonal; data security; digital exclusion etc)*
4. Overall, what feature or functions would you like to see in any future digital technologies developed to support care provision for people experiencing co-occurring heavy drinking and depression *(Probe:* signposting to appropriate services; access to NHS-approved apps; online information or advice)
5. Alternatively, what features or functions would not be useful or would cause you concerns? *(Probe:* unidentified source, access e.g. cost associated etc )

# Closing questions

1. Do you have any other thoughts or suggestions?

# End of interview

Thank participant for their time and switch off recorder.
