# Supplementary material for: Care professionals’ accounts of providing support and treatment for people with co-occurring alcohol use disorder and depression in the North East of England, UK: A qualitative study informed by complexity theory
Source: PLoS One. 2025 Oct 15;20(10):e0334524. doi: 10.1371/journal.pone.0334524 (PMC12527159; doi:10.1371/journal.pone.0334524)
Supplement: S3 File — (DOCX) [file pone.0334524.s003.docx]

**Supplementary information: *Broad thematic areas and codes generated from the full data and how these mapped to Complexity Theory-informed conceptual themes***
