# Supplementary material for: Care professionals’ accounts of providing support and treatment for people with co-occurring alcohol use disorder and depression in the North East of England, UK: A qualitative study informed by complexity theory
Source: PLoS One. 2025 Oct 15;20(10):e0334524. doi: 10.1371/journal.pone.0334524 (PMC12527159; doi:10.1371/journal.pone.0334524)
Supplement: Attachment — Submitted filename: Response to Reviewers.docx [file pone.0334524.s005.docx]

| **COMMENTS** | **RESPONSE** |
| --- | --- |
| **EDITOR** | |
| This is well-written manuscript that addresses an important and timely topic: care professionals accounts of providing support and treatment for people who have co-occurring alcohol use disorder and depression in one area in England. The value of the study is clearly articulated. The reviewers have raised important points to address in your revision, particularly around the role of complexity theory in the study (i.e. defining complexity theory and being clear how it informed your data collection and analysis) and an enhanced statement of author reflexivity. These are important considerations which I'd like to see addressed in the revised version. Some of the language from complexity theory was used throughout the results section (e.g. interconnections, adaptations), but it wasn't always entirely clear to me how complexity theory influenced the data analysis and results presentation. For example, the Discussion section contains a discussion of feedback loops, but these weren't evident within the Results. Similarly, the limitations has a brief mention of defining meaningful boundaries, but there is no previous discussion about the process of making boundary judgements. I hope these comments and those from the reviewers are helpful as you revise and I look forward to receiving a revised and strengthened manuscript. | We thank the Editor and the reviewers for their helpful feedback on our manuscript.  We appreciate the opportunity to strengthen our explanation of our complexity theory (CT) informed the analysis of our qualitative data, and to ensure we make explicit where we identified evidence of specific CT concepts in our results.  We also take on board your point around the need to describe how we defined and applied the boundaries of our CAS and have added this information accordingly. |
| 1. Please ensure that your manuscript meets PLOS ONE's style requirements, including those for file naming. The PLOS ONE style templates can be found at <https://journals.plos.org/plosone/s/file?id=wjVg/PLOSOne_formatting_sample_main_body.pdf> and https://journals.plos.org/plosone/s/file?id=ba62/PLOSOne_formatting_sample_title_authors_affiliations.pdf | We have edited and renamed files accordingly. |
| 2. In the online submission form, you indicated that the data that support the findings of this study are available on request from the corresponding author (AOD). The data are not publicly available due to their containing information that could compromise the privacy of research participants. All PLOS journals now require all data underlying the findings described in their manuscript to be freely available to other researchers, either a. In a public repository, b. Within the manuscript itself, or c. Uploaded as supplementary information. This policy applies to all data except where public deposition would breach compliance with the protocol approved by your research ethics board. If your data cannot be made publicly available for ethical or legal reasons (e.g., public availability would compromise patient privacy), please explain your reasons on resubmission and your exemption request will be escalated for approval. | In accordance with the terms of our ethical approval for this study, we are required to manage access to the full original interview transcripts. This is because the study took place in a relatively small geographical area, meaning we need to be especially careful about ensuring the anonymity and confidentiality of the service providers. For example, participants were assured that we would not use their verbatim quotes in any reporting if the content was likely to identify them and that only named members of the research team would have access to the transcripts. We hope this provides sufficient reasons for exemption. |
| 3. Please include captions for your Supporting Information files at the end of your manuscript, and update any in-text citations to match accordingly. Please see our Supporting Information guidelines for more information: <http://journals.plos.org/plosone/s/supporting-information>. | We have edited accordingly. |
| 4. Please remove all personal information, ensure that the data shared are in accordance with participant consent, and re-upload a fully anonymized data set. Note: spreadsheet columns with personal information must be removed and not hidden as all hidden columns will appear in the published file. | N/A |
| 5. If the reviewer comments include a recommendation to cite specific previously published works, please review and evaluate these publications to determine whether they are relevant and should be cited. There is no requirement to cite these works unless the editor has indicated otherwise. | N/A |
| **REVIEWER #1** | |
| The issue you address is of the utmost importance and studies like yours are definitely welcomed. But in its current form, this manuscript has two major flaws that needs to be fixed before we can assess its potential contribution. | We thank you for acknowledging the importance of this area of research and hope we can address your concerns – see below. |
| In this manuscript, you aimed at offering key insights on how health care and social care services are capable of dealing with the complex case of patients struggling with both alcohol use disorders and depression. The originality of your contribution being that you adopted a complexity theory perspective. This is the key feature of your paper that had me accepting to review it. Unfortunately, there is too little that is said on the complexity theory for your readers to know what your work is about. Having some good knowledge of the CT, I could have done without a description of it but the problem does not stop there. A major flaw in your manuscript is that you provide no clues on how the CT has informed data collection and analysis. These are the two main problems to which I add other minor ones that I summarise in the next few lines. | We appreciate the reviewer has existing expertise in complexity theory – however we feel a brief explanation is needed for readers less experienced in this area.  In relation to the reviewers next point – around a lack of clarity around how CT has informed data collection and analysis – we agree this is a weakness in the paper as it stands. We have therefore implemented several changes to the paper including adding more detail to the methods to describe how/when CT concepts were applied, incorporation (where appropriate) of more explicit CT language in the results section, and reflection on the limitations of our approach to using CT in the discussion. We hope this helps address your concerns. |
| I am under the impression that your abstract does not align well with what I found in the paper. | We are sorry the reviewer found a lack of alignment between the paper and the abstract – we have thoroughly reviewed the abstract in light of the revised manuscript and made a minor change. If the reviewer feels additional edits are needed, we would very much appreciate their specific feedback. |
| For the reporting of your results, I can see that you follow most of the guidelines one can find, for instance in COREQ, however, I feel you need to make more visible how your statements/ observations are rooted in your dataset. For instance, writing “some participants” or “several participants” instead of “X participants (P1, P2, …)” cast some doubts on the rigour put into the analyses. There are a number of statements/observations that lack any reference to the data that makes them legitimate. | We appreciate the reviewers feedback, but it is not usual practice to quantify perspectives collected via qualitative semi-structured interviews in this manner (i.e. whilst we focussed discussions around a broad set of questions, not all participants would be asked the same question in the same way, and some topics arose in some interviews but not others). We have added a sentence to the strengths and limitations section to remind readers that no inferences should be drawn regarding the prevalence of the issues/perspectives reported beyond our participant sample – as advised by Neale et al (see <https://onlinelibrary.wiley.com/doi/10.1111/add.12408>). Elsewhere, we have ensured that the language used to highlight commonality or otherwise in viewpoints avoids inappropriate language, again in line with recommended best practice (e.g. some/many as opposed to most). |
| The use of acronyms is a bit of a pain, especially for your readers based outside UK. They should be avoided as much as possible. In table 1, for instance, I had to go back to the text to get what was the NENC ICS standing for. I also struggled with the VCSE. | We appreciate this point, particularly considering international readers. We have reviewed use of acronyms throughout and endeavoured to employ more judiciously or write out in full as suggested. For example, NENC ICS is now written in full throughout; AUD remains abbreviated as this is a commonly used acronym internationally. |
| I would suggest that instead of writing “Participant X”, when quoting your interviews, you use a short form like PX. The long form should be for the type of professional interviewed. | We have made this change as requested throughout as appropriate. |
| On page 5, line 68, I do not understand what you mean by: “particularly in countries with insurance-based health systems (16, 17).” | We have edited this to now read ‘…particularly in countries without universal health care’ (p5 line 70). |
| Page 11, line 215: “1) one researcher (KJ) familiarized themselves”… themselves or herself? | We have revised to ‘herself’ (recognising that we provide gender elsewhere in the methods). (p12 line 251) |
| Page 25, line 502: “there is a large extant literature”… check whether this is the right way to say it. | We have revised to ‘existing’ literature (p28 line 611). |
| To sum up my review, it is by far the problem of the application of the complexity theory to this topic that is most problematic. To be honest, I feel cheated having seen in the title reference to this theory but with nothing in the text that really makes it up to this promise. | We are disappointed to receive this feedback but hope that the changes implemented above will resolve the reviewer’s concerns. |
| **REVIEWER #2** | |
| This is an interesting and well described study. The study provides important insights into service provision for people with co-occurring AUD and depression. The use of complexity theory to understand the results is a key strength of the research. | We thank the reviewer for their positive feedback. |
| Line 154. Is there a reference for the larger background study? | Whilst there is no published protocol, we have provided a reference to the funder’s website that we hope is helpful. See p9 line 158. |
| Line 179: For consistency, can you provide a number for the ‘wide range’ of services that provide support for AUD and mental health? | As these services change almost constantly due to short term funding and commissioning changes (which is indeed a substantial barrier to effective care) it is difficult to provide a meaningful number here – additionally, as mentioned above, due to the relatively small size of the case study region, we are reluctant to name specific local organisations in order to preserve anonymity etc. We have tried to instead provide some examples of national organisations that operate in the case study region and hope this is helpful/acceptable. See p10 lines 188 to 195. |
| Section 2.2: Please state the number of participants the study aimed to recruit | We have added this information to our methods section (see p11 line 213) |
| Section 2.3: Please state if individuals were individual or focus group interviews | All interviews were individual (one-to-one) and conducted online or in-person, according to participant preference. We have updated the methods to make this clear – see p12 line 240. |
| Section 2.4: Please provide further detail in relation to how the coding framework was developed. This section states that all data were coded and then the coding framework was developed and applied to the data. If the data was already coded, why was the coding framework needed? What purpose did this serve? | Apologies and thank you for highlighting that this was not clear. We have edited this section so it is hopefully clearer when the coding framework was developed and applied. See p12. |
| Table 1 presents information on participant characteristics, the methods section states that recruitment was targeted to different professional roles. Including information on the role of participants is likely to be useful to the reader and help contextualise the findings - but, including this information needs to be balanced against the likelihood of possible jigsaw identification. | Thank you – as the reviewer acknowledges, and as emphasised elsewhere, to protect the anonymity of our participants, we identify interview subjects by job sector only (or in the case of one quote where we feel a participant might be identifiable by job sector, we removed all identifiers). |
| Discussion: Line 540: The section on peer support in recovery may be better placed in the implications section. Further discussion would be of benefit here to provide information on how peer services are being utilsed more broadly in the AUD / mental health treatment space. | Thank you for this suggestion – we have moved and expanded this section accordingly, see p33 and 35. |
| Discussion: Consideration and discussion of how the results of the present study relate to the role of the ICS would be of use. Specifically, what implications do the results have for ICS service provision? | We have expanded this section to consider the implications in relation to the ICS – see p34. |
| Lastly, the study uses reflexive thematic analysis, as such, a detailed reflexivity statement is needed. Some detail on the two researchers involved in data coding is presented in the methods section, but additional information is needed re how the researchers’ prior knowledge and experience may have influenced or shaped the research. This could either be situated in the method or discussion section. | Thanks for pointing out this oversight – we have added some further content addressing this in the methods – see ps12-13. |
